# Social identity processes associated with perceived risk at pilot sporting events during COVID‐19

**DOI:** 10.1111/bjso.12541

**Published:** 2022-04-15

**Authors:** Kayleigh Smith, Anne Templeton

**Affiliations:** ^1^ Department of Psychology University of Edinburgh Edinburgh UK

**Keywords:** COVID‐19, event planning, event safety, group processes, mass events, perceived risk

## Abstract

Previous research suggests that shared social identification and expected support from others can reduce the extent to which attendees of mass events perceive that others pose health risks. This study evaluated the social identity processes associated with perceived risk at UK pilot sporting events held during COVID‐19, including the government Events Research Programme. An online survey (*N* = 2029) measured attendee perceptions that other spectators adhered to safety measures, shared social identity with other attendees, expectations that others would provide support, and the perceived risk of germ spread from other attendees. Results indicate that for football attendees, seeing others adhering to COVID‐19 safety measures was associated with lower perceived risk and this was partially mediated via increased shared social identity and expected support. However, the sequential mediations were non‐significant for rugby and horse racing events. The decreased perceived risk for football and rugby attendees highlights the importance of understanding social identity processes at mass events to increase safety. The non‐significant associations between shared social identity and perceived risk and between expected support and perceived risk for both the rugby and the horse racing highlights the need to further research risk perceptions across a range of mass event contexts.

## BACKGROUND

On 16 March 2020, all mass events in the United Kingdom were halted due to increasing concern of COVID‐19 spread (UK Government, 2020a). The potential spread of disease at events is a prominent health concern at mass events (Karami et al., 2019). This is in part due to issues such as close proximity between large numbers of attendees (e.g. see Benkouiten et al., 2013; Memish et al., 2014), as well as travel to and from the event and gatherings around the event itself (e.g. see Smith et al., 2021). However, mass events can also provide positive benefits to attendees, such as improved mental health (Cruwys et al., 2019), reduced stress and increased positive emotions (Hopkins & Reicher, 2017). They also provide substantial economic benefits. For example, the Events Research Programme Phase 1 report highlighted the significant contribution that spectator sporting provided to the English economy pre‐covid (Department for Digital, Culture, Media, & Sport, 2021). The return of spectator sporting, therefore, poses risks for COVID‐19 transmission but can be beneficial for both the spectators themselves and the broader economy.

Protective health behaviours such as physical distancing can mitigate the spread of COVID‐19 at mass events, but this requires attendees to understand the risks associated with their behaviour and follow the guidance accordingly. A range of pilot sporting events have been held throughout the COVID‐19 pandemic to explore the risk of reopening mass events, including those initiated by the UK government and led by UK Sport and the Sports Grounds Safety Authority between July and September 2020, and the UK Government's Events Research Programme running from April to July 2021. These events were held to evaluate whether spectators could safely return to sporting events with (and eventually without) COVID‐19 safety measures in place, including the wearing of facemasks, physical distancing, increased handwashing/sanitizing and limited occupancy of events. As countries move towards allowing large mass events to reopen, it is important to understand what factors are associated with attendee perceptions of risk to encourage safe behaviour during event reopening.

Risk perception and shared social identity have both been found to be significant predictors of ticket purchasing for sporting events (Silveira et al., 2018). Initial reports from the Events Research Programme show that spectators felt safe and perceived low risk of COVID‐19 spread across the events (Templeton et al., 2021). However, recent evidence from Public Health England demonstrates that these events were associated with an increase in COVID‐19 cases (although, importantly, this was partially due to mixing outside of the events; Smith et al., 2021). The incompatibility between perceived and actual risk, as well as the necessity for attendees to understand risk in order to act safely, shows it is crucial to explore the variables associated with attendees’ perceived risk at mass events.

In addition to shared social identities being a reason for event attendance, it is also a key predictor of perceived risk of disease spread. Previous research on the role of social identity processes in perceived risk suggests that people may perceive members of their group as posing less risk (Cruwys et al., 2021), or they may have less concern about the risk posed by members of their group (e.g. see Khazaie & Khan, 2019). As such, social identity processes can provide a vehicle to understanding how and why attendees of large events interact with one another in potentially high‐risk events such as mass sporting events. This study aims to evaluate the effect of social identity processes on perceived risk at four sporting events that took place in the United Kingdom in 2020 and 2021 during the COVID‐19 pandemic.

### Association between observing others’ adherence and reduced risk perception

Safety measures such as physical distancing and the wearing of facemasks were in place to increase the safety of events. Indeed, research has demonstrated the effectiveness of physical distancing and facemasks in reducing the spread of COVID‐19 (Chu et al., 2020). YouGov polls from July 2021 demonstrate that most Britons believed in these protective health behaviours, since the majority of respondents found hand washing/sanitizing, face mask wearing and physical distancing to be either effective or very effective in preventing the spread of COVID‐19 (YouGov, 2021). The awareness of reduced risk when engaging in these protective health behaviours suggests that observing others adhering to the safety measures may also result in lower perceived risk of COVID‐19 spread. It is, therefore, hypothesized that seeing others’ adhering to safety measures will be associated with lowered perceptions of risk (*H1*).

### Adherence to COVID‐19 guidance as an indicator of normative fit

Mass events provide an environment where a physical crowd—a collection of individuals who happen to be in the same place at the same time without a meaningful social connection between them—can become a psychological crowd—where the individuals in the shared space perceived each other as being part of a shared social category (Reicher, 2011). The social identity approach—comprising of social identity theory (Tajfel & Turner, 1979) and self‐categorization theory (Turner et al., 1987)—provides a theoretical framework for understanding how attendees come to perceive one another as fellow group members, and crucially how to evaluate the role of social identity processes on perceived risk.

Social identity theory posits that people have multiple social identities, whereby each social identity has its own norms and values associated with it (such as the norms of English compared to Scottish football fans; Stott et al., 2001). Mass events can have their own established norms, and these can have both positive and negative consequences. For example, it is normative at the Magh Mela for attendees to provide support and help to others (positive consequence), but it is also normative for pilgrims to endure harsh and dangerous weather conditions which can result in illness (negative consequence; Pandey et al., 2014). Norms and values are flexible and dynamic, such that behaviours that were once non‐normative (such as actions against the police) can shift to being normative (protestors pushing back against the police) if the context changes (Stott & Drury, 2000). Self‐categorization theory highlights how individuals can shift from their personal identity to a social identity through processes such as self‐stereotyping and depersonalisation. At mass events, individuals can incur a cognitive transformation from their personal identity to a social identity where they perceive themselves as being part of a group with the others at the event (Reicher, 2012). Once the social identity is salient, members can adopt the values, goals and beliefs of the group (Reicher, 2011).

The social identity approach shows how observing behaviours that are typical of the salient social category—that is, perceiving normative fit—can indicate group membership and increase a sense of shared social identity with fellow group members (Turner et al., 1987). This has important implications for mass events during COVID‐19. Previous surveys with spectators of the 2020 pilot sporting events showed that attendees were highly motivated to adhere to the COVID‐19 safety guidance to keep other spectators safe and to enable the events to reopen (Templeton et al., 2020). Similarly, interviews with attendees of 2021 Event Research Programme events indicated that attendees perceived adherence to the safety guidance as a shared group goal to support the Programme opening larger events in the future (Templeton et al., 2021). In this context, following the safety guidance was seen as a method to support the group and an indicator of group membership. Thus, observing other spectators adhering to safety measures was associated with increased perceived shared social identity as it symbolized belonging to the group and demonstrated providing support and care for others alongside acting normatively in line with group goals and beliefs. It is, therefore, hypothesized that perceiving others adhering—thus demonstrating normative fit—will be associated with an increase in shared social identity experienced by spectators at the pilot events (*H2*).

### Group processes associated with attenuated perceived risk

Previous research suggests that increased shared social identity can be associated with seeing in‐group members as less risky than outgroup members, for both general risks (e.g. Cruwys et al., 2021) and risk of contracting disease (e.g. Cruwys et al., 2021; Khazaie & Khan, 2019). For example, Khazaie and Khan (2019) compared high and low shared social identity salience in perceptions of health risks, including vulnerability to disease, likelihood to engage in health risk behaviours and the perceived risk associated with these health risk behaviours. They found that participants with high shared social identity in both laboratory settings and at UK music festivals were found to perceive lower vulnerability to disease and greater likelihood to engage in health risk behaviours than those with low shared social identity.

Similarly, Cruwys et al. (2021) ran a series of studies which consistently show how in‐group membership can attenuate perceived risk. They found that participants perceived a lower risk of disease from in‐group members compared with outgroup members, particularly when there was a high salience of disease risk. Moreover, in a cross‐sectional survey with festival attendees, they found that greater shared social identity was associated with reduced perceptions of risk. Together, the series of studies highlight the replicability of this effect across a range of contexts, where increased shared social identity reduced perceptions of risk both specific to disease and risk more generally.

Based on previous research that increased shared social identity is associated with reduced risk perception, we further hypothesize that the relationship between seeing others’ adhering to COVID‐19 safety guidance and perceived risk that others can spread germs will be mediated by shared social identity with others at the pilot sporting events (*H3*).

### Expecting support from the group to keep safe

Increased shared social identity can lead to increased cooperation, trust, agreement and support between group members (Reicher, 2012). This effect has been demonstrated across a range of mass events. For example, Pandey et al. (2014) evaluated expected support at the 2010 Magh Mela, an annual Hindu religious festival held during the winter where pilgrims live in basic conditions and bathe in the freezing Ganges each morning. Through interviewing attendees, they found that greater helping behaviour was both expected and received from those who were considered to be in‐group members. Pilgrims described knowing that they would be provided care and support if they required it (such as if they fell ill) from those with whom they had a shared social identity (Hopkins & Reicher, 2017). These findings can also be seen in mass emergencies. For example, residents involved in the 2015–2016 York floods reported greater expectations of support both during and after the floods when they had a shared social identity with other victims (Ntontis et al., 2018, 2020). In line with these findings, it is hypothesized that shared social identification with attendees at the sporting events will be positively associated with expected support to keep safe (*H4a*).

Expected support has also been shown to increase through observing the supportive behaviours of others. For example, Drury et al. (2016) surveyed victims of the 2010 Chile earthquakes to evaluate the influence of observing emotional and behavioural social support on expectations of support. They found that observing emotional support, such as seeing others showing respect for each other or showing concern for the needs of other victims increased the support expected from in‐group members. Additionally, they found that observing behavioural support—such as seeing others’ working together to respond to the earthquake or seeing others’ sharing resources—increased expectations of support. Similarly, Ntontis et al. (2018) found that observing supportive behaviours, such as seeing others’ providing helping behaviour, increased interviewees’ expectations of support both during the emergency and in any future emergencies. Although the prior literature comes from community emergencies, it provides a basis to evaluate the relationship between observing supportive behaviour on expected support in other mass event contexts. In the present COVID‐19 context, supportive behaviours such as adhering to COVID‐19 safety measures are hypothesized to be associated with increased expectations that other attendees will provide support to others to keep safe (*H4b*).

Previous research also suggests that an increase in expected support from group members can reduce perceived risk at mass events. For example, Alnabulsi and Drury (2014) found that a greater shared social identity with others at the Hajj indirectly increased perceptions of safety via an increase in expected support from in‐group members, even in a highly dense and potentially dangerous environment. Similarly, Drury et al. (2015) evaluated perceived risk at the Big Beach Boutique II party at Brighton beach in 2002, another highly dense mass event that potentially posed a significant risk to attendees. They found that increased identification with the crowd increased perceptions of safety via the expectation that others would provide help if it were required. Together, these studies demonstrate that shared social identity was associated with an increase in expected support and reduced general perceptions of risk at different mass events.

It is clear from the prior literature that there are established associations between observing supportive behaviour and expected support, which in turn has been associated with reduced perceptions of risk. Thus, it is hypothesized that the relationship between others’ adherence and perceived risk will be mediated by expected support (*H4c*). Moreover, observing supportive behaviour has been associated with increased shared social identity, which in turn has been associated with increased expectations of support, which then has demonstrated associations with reduced risk perception. As such, we hypothesize that there will be a sequential mediation wherein seeing others adhere to the COVID‐19 safety guidance will be associated with reduced perceived risk via increased shared social identity and increased expected support to keep safe (*H4d*).

### Present study

**FIGURE 1:**
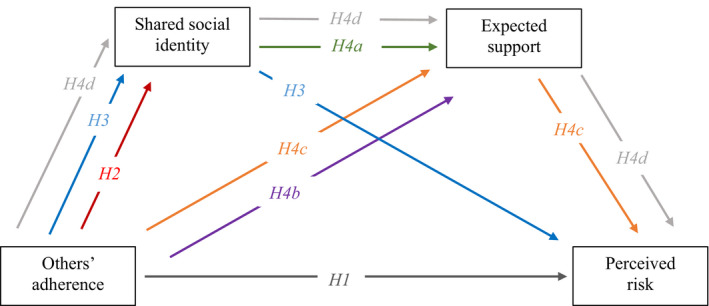
Hypothesized mediation models

The COVID‐19 pandemic has emphasized the importance of understanding the factors associated with risk perception, particularly when considering the potential behavioural implications including risk‐taking behaviour (Hopkins & Reicher, 2017) alongside the impact of risk perception on attendance at spectator events (Silveira et al., 2018). The present context allows us to evaluate the theory in the extreme and unique context of the COVID‐19 pandemic. The present study aims to expand the prior literature by evaluating the role of seeing other spectators adhering to safety measures, shared social identity and support expected from in‐group members on spectator perceived risk at the pilot sporting events held during COVID‐19. This shall be evaluated across three sports—football, rugby and horse racing—to evaluate whether the model holds in different sporting environments (i.e. open‐air theatre and open air). Different environments pose varying levels of COVID‐19 transmission risk, as demonstrated in the Events Research Programme (Department for Digital, Culture, Media, & Sport, 2021) where varying levels of co2 emissions were found in different environments (co2 being a risk indicator of COVID‐19 spread). Spectators also demonstrated awareness of the differing risks between environments, where outdoor environments were perceived to be of lower risk (Templeton et al., 2021). This highlights the importance of evaluating whether this mediation model holds in each of these environmental contexts. Moreover, we include pilot sporting events that took place within the United Kingdom in both 2020 and 2021 to evaluate whether the model holds across different time points in the pandemic. A summary of the model and all hypotheses are shown in Figure 1.

## METHODS

### Ethics and open science statement

This study was approved by the [University of Edinburgh] Ethics Committee (reference 397‐1920/4 and reference 397‐1920/6). A full list of scale items used in this study can be found in the Supplementary Materials and on the Open Science Framework (https://osf.io/25yw4/?view_only=9b8ff3e70add460f9436fe6244be9604).

### Participants

For the pilot sporting events in 2020, a total sample of 2326 responses were collected from attendees. Participants were excluded if they did not provide consent (*N* = 51), complete the demographic questions (*N* = 160), report which event they attended (*N* = 276) or completed the survey more than 15 days after the event (*N* = 18). The total final sample was 1821. Three events from across the September 2020 pilot sporting events were considered in this analysis. These included the Football League One event (going forward named football event 1; *N* = 273), a Harlequins versus Bath rugby match (going forward named rugby; *N* = 252) and the horse racing at St Leger (going forward named horse racing; *N* = 175; although this event was scheduled for multiple days, it only held spectators for 1 day due to rising COVID‐19 cases and the concerns associated (Keogh, 2020)).

For the 2021 football cup final held in May 2021 during the Events Research Programme (going forward named football event 2), a total sample of 1800 responses were collected from attendees. Participants were excluded for failing the attention check (*N* = 167), not completing the demographic questions (*N* = 298), and for being outliers (*N* = 6). All participants completed the survey within 10 days of attending the event. The total final sample was 1329.

The demographic information (age, gender, employment status and home region) for participants included in the analysis can be found in Table 1.

**TABLE 1:** Participant demographic information

Demographics	Football event 1		Rugby		Horse racing		Football event 2	
	Frequency	%	Frequency	%	Frequency	%	Frequency	%
Gender								
Male	218	79.85	185	73.41	112	64	1004	75.54
Female	54	19.78	65	25.79	62	35.43	320	24.08
Transgender female	0	0	0	0	0	0	2	0.15
Did not disclose	1	0.37	2	0.79	1	0.57	3	0.23
Age								
18–24	13	4.76	8	3.17	4	2.29	44	3.31
25–34	30	10.99	25	9.92	14	8	155	11.66
35–44	32	11.72	37	14.68	22	12.57	164	12.34
45–54	39	14.29	75	29.76	37	21.14	247	18.59
55–64	81	29.67	72	28.57	65	37.14	481	36.19
65–74	73	26.74	31	12.30	25	14.29	218	16.40
75–84	3	1.10	3	1.19	7	4	20	1.51
Over 85	1	0.37	0	0	0	0	0	0
Did not disclose	1	0.37	1	0.40	1	0.57	0	0
Home region								
England	273	100	252	100	170	97.14	1319	99.25
Scotland	0	0	0	0	3	1.72	3	0.23
Wales	0	0	0	0	2	1.14	6	0.45
Outside UK	0	0	0	0	0	0	1	0.07
Employment status								
Employed full‐time	138	50.55	174	69.05	83	47.43	854	64.26
Employed part‐time	19	6.96	14	5.56	20	11.43	113	8.50
Unemployed but looking for work	5	1.83	6	2.38	3	1.71	12	0.90
Unemployed but not looking for work	4	1.47	1	0.40	2	1.14	8	0.60
Retired	99	36.26	52	20.63	59	33.71	302	22.72
Student	3	1.10	0	0	3	1.71	19	1.43
Disabled	2	0.73	2	0.79	1	0.57	6	0.45
Furloughed	1	0.37	2	0.79	2	1.14	3	0.23
Full‐time carers	2	0.73	1	0.40	2	1.14	2	0.15
Did not disclose	0	0	0	0	0	0	10	0.75

### Measures

A correlational study design was adopted, using a survey to evaluate the relationship between others’ adherence, shared social identity, expected support and perceived risk. The scales were measured using a 7‐point Likert scale for the 2020 events and a 5‐point Likert scale for the football cup final match held in May 2021. Item one of the shared social identity scale (‘I thought that everyone in the crowd felt part of the same group’) was added by the researchers to fit the context of the events, item two was taken adapted from Doosje et al. (1995), and item three was adapted from Leach et al. (2008). The expected support measure was adapted from Alnabulsi and Drury (2014; items one and three) and Alnabulsi et al. (2018; item 2). Perceived risk measures were adapted from Cruwys et al. (2021). Others’ adherence measures were developed by the researchers based on the COVID‐19 safety measures attendees were asked to follow. Example questions for each scale are presented in Table 2.

**TABLE 2:** Scale items, reliability per event and means/standard deviations

Scale	Example item	Football event 1		Horse racing		Rugby		Football event 2	
		*α*	*M* (*SD*)	*α*	*M* (*SD*)	*α*	*M* (*SD*)	*α*	*M* (*SD*)
Others’ adherence (three items)	‘To what extent do you disagree or agree that the other attendees overall adhered to the following safety measures – physical distancing’	.82	5.981 (*1*.*015*)	.79	5.992 (*0*.*994*)	.72	5.541 (*0*.*979*)	.82	3.064 (*1*.*072*)
Shared social identity (three items)	‘I thought everyone in the crowd felt part of the same group’	.80	6.042 (*0*.*969*)	.78	5.303 (*1*.*291*)	.80	5.677 (*1*.*006*)	.92	3.978 (*0*.*949*)
Expected support (three items)	‘I felt that the other crowd members took care of one another’	.89	5.515 (*1*.*114*)	.89	5.459 (*1*.*227*)	.86	5.103 (*1*.*074*)	.87	3.052 (*0*.*883*)
Perceived risk (two items)	‘I was concerned about other crowd members spreading germs’	*r* = .68	2.597 (1.383)	*r* = .65	2.457 (*1*.*374*)	*r* = .64	2.940 (*1*.*344*)	*r* = .69	2.705 (*1*.*090*)

The questions regarding others’ adherence asked about the key COVID‐19 safety measures in place at the events: washing hands/sanitizing regularly, keeping physically distant from other attendees and wearing a face mask as part of the COVID‐19 mitigating measures.

### Procedure

Participants had all attended either one of the UK trial sporting events held in September 2020, or a football cup final in May 2021, where the present study was advertised both at the events and to ticket holders after attending. Spectators were invited to participate in an online survey via Qualtrics. After obtaining consent, participants were presented with items related to others’ adherence, followed by shared social identity, then expected support and perceived risk, and finally by the demographic questions before being debriefed. The survey took participants 10–15 min to complete. To ensure responses were not influenced by a fading affect bias—where the negativity of an experience (e.g. perceived risk) tends to be reported as less negative as the time from the experience increases (Ritchie et al., 2015)—a cut‐off date for participation was used. Although prior research has used a 4‐week cut‐off (Khazaie & Khan, 2019), a more conservative cut‐off of 15 days after the event was adopted because UK government guidance at the time highlighted that any symptoms of COVID‐19 would show within 14 days, and a lack of symptoms could influence responses of risk perception. No monetary reward was offered for participation.

### Data analysis plan

The R programming language version 4.0.3 (R Core Team, 2020) was used for all analyses. An exploratory factor analysis with eigen rotation (direct oblimin) was carried out using the psych package version 2.0.12 (Revelle, 2020) to ensure all items were clustering onto the appropriate factors. Descriptive statistics, including the correlations between the variables, were obtained. The Lavaan package version 0.6.8 (Rosseel, 2012) was used to perform a mediation analysis using 95% bias‐corrected confidence intervals. As the assumption of normality of residuals was violated, all mediation analyses were conducted using 10,000 standard error bootstrapped samples. This model provided the regressions between each variable in the model, the direct effects, the simple mediations via each mediator and the sequential mediation via both mediators. In this model, the independent variable (X) was others’ adherence, the outcome variable (Y) was perceived risk, mediator one (M1) was shared social identity, and mediator two (M2) was expected support. An alpha level of 05 was used for all statistical tests.

## RESULTS

### Factor analysis

An exploratory factor analysis was conducted on the 14 items across all scales with eigen rotation (direct oblimin) for each individual event. The Keiser–Meyer–Olkin (KMO) verified the sampling adequacy for the analysis, where all KMO values were above .66 for all four events, above the limit of .5 recommended by Field et al. (2012). An initial analysis was run to obtain eigenvalues for each factor. Four factors had eigenvalues over Kaiser's criterion of 1, and in combination these explained 66%–72% of the variance across the four events. These four factors were retained in this analysis. The item clustering suggests that factor one represents shared social identity, factor two represents expected support, factor three represents perceived risk, and factor four represents others’ adherence.

For both rugby and football event 2, all items clustered onto the factors as expected. However, for football event 1 and the horse racing, one item from perceived risk (‘I felt safe when I was with other crowd members’) was also highly grouped into the expected support factor. This item was dropped from all events to avoid covariance in the model and to improve scale reliability.

### Descriptive statistics

Table 3 provides descriptive statistical information regarding the means and standard deviations of each variable at each event. In addition, it highlights the correlations between all the continuous variables used for this analysis.

**TABLE 3:** Means, standard deviations and correlations for all events

Variable	Mean	*SD*	2.	3.	4.
Football event 1					
1. Others’ adherence	5.981	1.015	.479***	.596***	−.443***
2. Shared social identity	6.042	0.969	–	.538***	−.347***
3. Expected support	5.515	1.114	–	–	−.458***
4. Perceived risk	2.597	1.383	–	–	–
Horse racing					
1. Others’ adherence	5.992	0.994	.435***	.679***	−.302***
2. Shared social identity	5.303	1.291	–	.588***	−.210***
3. Expected support	5.459	1.227	–	–	−.274***
4. Perceived risk	2.457	1.374	–	–	–
Rugby					
1. Others’ adherence	5.541	0.979	.467***	.589***	−.375***
2. Shared social identity	5.677	1.006	–	.513***	−.299***
3. Expected support	5.103	1.074	–	–	−.317***
4. Perceived risk	2.940	1.344	–	–	–
Football event 2					
1. Others’ adherence	3.064	1.072	.249***	.575***	−.319***
2. Shared social identity	3.978	0.949	–	.386***	−.274***
3. Expected support	3.052	0.883	–	–	−.424***
4. Perceived risk	2.705	1.090	–	–	–

* *p* < .05; **p < .01; ***p < .001.

### Regression analysis

Table 4 demonstrates the individual regressions between each of the variables in this analysis for the four events.

**TABLE 4:** Regressions

	*b*	LCI	UCI	SE	*z*	*p*
Football event 1						
OA–SSI	0.479	0.275	0.630	0.092	4.995	<.001
OA–ES	0.440	0.289	0.666	0.098	4.943	<.001
OA–PR	−0.244	−0.632	−0.106	0.135	−2.468	.014
SSI–ES	0.326	0.226	0.545	0.081	4.617	<.001
SSI–PR	−0.082	−0.361	0.090	0.115	−1.020	.308
ES–PR	−0.267	−0.586	−0.102	0.124	−2.659	.008
Horse racing						
OA–SSI	0.435	0.388	0.775	0.098	5.795	<.001
OA–ES	0.522	0.471	0.804	0.084	7.621	<.001
OA–PR	−0.212	−0.635	0.067	0.179	−1.635	.102
SSI–ES	0.361	0.207	0.496	0.075	4.586	<.001
SSI–PR	−0.063	−0.320	0.128	0.113	−0.595	.552
ES–PR	−0.092	−0.365	0.166	0.135	−0.767	.443
Rugby						
OA–SSI	0.467	0.352	0.609	0.066	7.313	<.001
OA–ES	0.446	0.365	0.615	0.064	7.675	<.001
OA–PR	−0.256	−0.568	−0.127	0.112	−3.151	.002
SSI–ES	0.308	0.202	0.464	0.066	4.964	<.001
SSI–PR	−0.111	−0.328	0.035	0.093	−1.596	.111
ES–PR	−0.115	−0.354	0.067	0.108	−1.339	.181
Age–PR	−0.095	−0.206	0.008	0.055	−1.745	.081
Football event 2						
OA–SSI	0.254	0.177	0.286	0.025	8.880	<.001
OA–ES	0.512	0.382	0.461	0.020	20.844	<.001
OA–PR	−0.112	−0.178	−0.049	0.033	−3.505	<.001
SSI–ES	0.257	0.196	0.281	0.022	11.003	<.001
SSI–PR	−0.124	−0.210	−0.079	0.033	−4.267	<.001
ES–PR	−0.309	−0.462	−0.296	0.043	−8.854	<.001
Age–SSI	−0.061	−0.081	−0.003	0.020	−2.088	.037
Employment–SSI	0.058	0.001	0.056	0.014	2.054	.040
Age–ES	−0.044	−0.055	0.001	0.014	−1.927	.054
Age–PR	0.073	0.018	0.096	0.020	2.902	.004

Note

*b* = standardized beta coefficients; LCI =lower confidence interval; UCI =upper confidence interval; SE =standard error; OA =others’ adherence; SSI =shared social identity; ES =expected support; PR =perceived risk. All demographic variables were tested for significant associations with shared social identity, expected support and risk perception using standard error bootstrapped regressions with 10,000 samples. Only the significant demographics are included in the table and structural models.

### Mediation analysis

**FIGURE 2:**
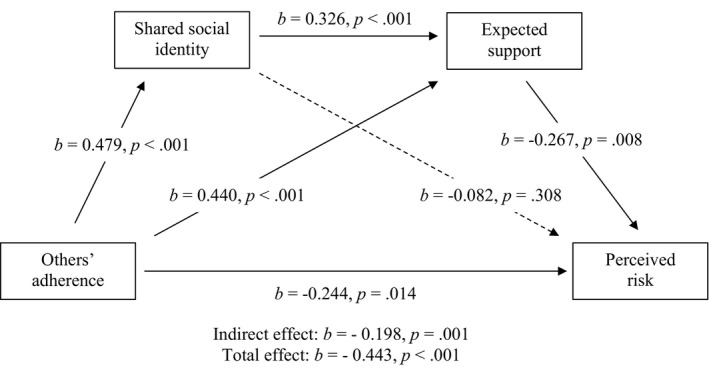
Sequential mediation model for football event 1

**FIGURE 3:**
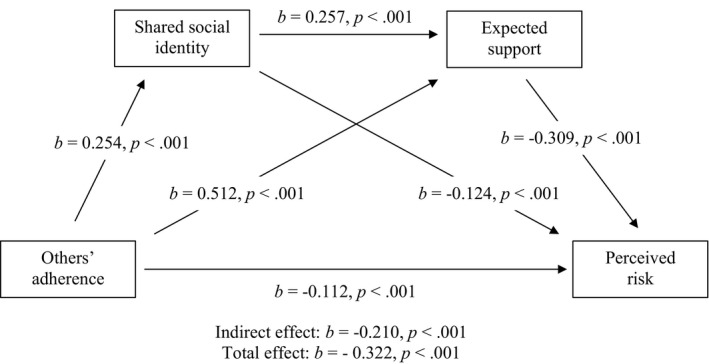
Sequential mediation model for football event 2

**FIGURE 4:**
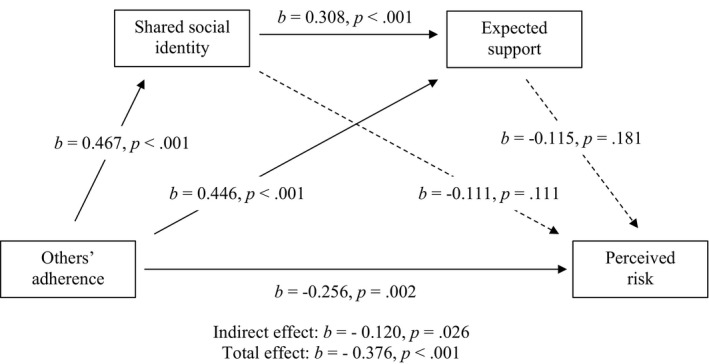
Sequential mediation model for rugby

**FIGURE 5:**
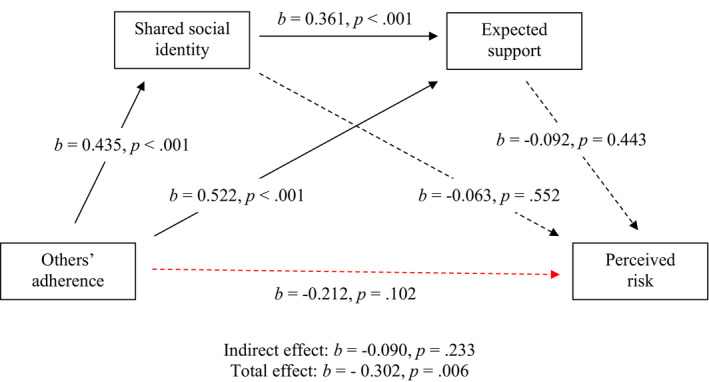
Sequential mediation model for horse racing

Full tables and visualizations of results can be found at the end of the results (football event 1: Table 5, Figure 2; football event 2: Table 6, Figure 3; rugby: Table 7, Figure 4; horse racing: Table 8, Figure 5).

**TABLE 5:** Total, direct and indirect effects for mediation analysis for football event 1

	*b*	LCI	UCI	SE	*z*	*p*
Total effect	−0.443	−0.792	−0.372	0.105	−5.730	<.001
Direct effect	−0.244	−0.632	−0.106	0.135	−2.468	.014
Indirect effects						
Total indirect effect	−0.198	−0.428	−0.117	0.078	−3.481	.001
OA–SSI–PR	−0.039	−0.174	0.043	0.054	−0.998	.318
OA–ES–PR	−0.117	−0.304	−0.058	0.061	−2.612	.009
OA–SSI–ES–PR	−0.042	−0.123	−0.019	0.025	−2.246	.025

Note

*b* = standardized beta coefficients; ES, expected support; LCI, lower confidence interval; OA, others’ adherence; PR, perceived risk; SE, standard error; SSI, shared social identity; UCI, upper confidence interval.

**TABLE 6:** Total, direct and indirect effects for mediation analysis for football event 2

	*b*	LCI	UCI	SE	*z*	*p*
Total effect	−0.322	−0.383	−0.270	0.029	−11.392	<.001
Direct effect	−0.112	−0.178	−0.049	0.033	−3.505	<.001
Indirect effects						
Total indirect effect	−0.210	−0.254	−0.173	0.021	−10.245	<.001
OA–SSI–PR	−0.032	−0.051	−0.017	0.008	−3.782	<.001
OA–ES–PR	−0.158	−0.200	−0.124	0.019	−8.264	<.001
OA–SSI–ES–PR	−0.020	−0.029	−0.014	0.004	−5.378	<.001

Note

*b* = standardized beta coefficients; ES, expected support; LCI, lower confidence interval; OA, others’ adherence; PR, perceived risk; SE, standard error; SSI, shared social identity; UCI, upper confidence interval.

**TABLE 7:** Total, direct and indirect effects for mediation analysis for rugby

	*b*	LCI	UCI	SE	*z*	*p*
Total effect	−0.376	−0.684	−0.335	0.088	−5.852	<.001
Direct effect	−0.256	−0.568	−0.127	0.112	−3.151	.002
Indirect effects						
Total indirect effect	−0.120	−0.316	−0.022	0.074	−2.221	.026
OA–SSI–PR	−0.052	−0.175	0.014	0.048	−1.493	.136
OA–ES–PR	−0.051	−0.186	0.030	0.054	−1.305	.192
OA–SSI–ES–PR	−0.017	−0.062	0.008	0.017	−1.318	.188

Note

*b* = standardized beta coefficients; ES, expected support; LCI, lower confidence interval; OA, others’ adherence; PR, perceived risk; SE, standard error; SSI, shared social identity; UCI, upper confidence interval.

**TABLE 8:** Total, direct and indirect effects for mediation analysis for horse racing

	*b*	LCI	UCI	SE	*z*	*p*
Total effect	−0.302	−0.723	−0.131	0.153	−2.740	.006
Direct effect	−0.212	−0.635	0.067	0.179	−1.635	.102
Indirect effects						
Total indirect effect	−0.090	−0.330	−0.082	0.104	−1.193	.233
OA–SSI–PR	−0.027	−0.185	0.073	0.065	−0.584	.559
OA–ES–PR	−0.048	−0.234	0.104	0.086	−0.775	.438
OA–SSI–ES–PR	−0.014	−0.082	0.032	0.028	−0.708	.479

Note

*b* = standardized beta coefficients; ES, expected support; LCI, lower confidence interval; OA, others’ adherence; PR, perceived risk; SE, standard error; SSI, shared social identity; UCI, upper confidence interval.

Hypothesis 1 Seeing others adhere to the COVID‐19 safety measures will be associated with reduced perceptions that other attendees pose risk of germ spread.

In support of *H1*, there was a significant direct effect of others’ adherence on perceived risk, where increased perceptions of others’ adhering to safety measures was associated with reduced perceptions of risk at football event 1 (*b* = −0.244, *p* = .014), football event 2 (*b* = −0.112, *p* < .001) and rugby (*b* = −0.256, *p* = .002). However, there was no significant effect of others’ adherence on perceived risk at the horse racing (*b* = −0.212, *p* = .102), not supporting *H1*.

Hypothesis 2 Seeing others adhere to the COVID‐19 safety measures will be associated with increased perceived shared social identity among attendees.

In support of *H2*, there was a significant effect of others’ adherence on shared social identity, where increased perceptions of others’ adhering to safety measures was associated with increased perceived shared social identity among attendees at football event 1 (*b* = 0.479, *p* < .001), football event 2 (*b* = 0.254, *p* < .001), the rugby (*b* = 0.467, *p* < .001) and the horseracing (*b* = 0.435, *p* < .001).

Hypothesis 3 The relationship between seeing others adhering to COVID‐19 safety measures and perceived risk will be mediated by shared social identification with other Attendees.

In support of *H3*, there was a significant indirect effect of others’ adherence on reduced perceived risk via increased shared social identity at football event 2 (*b* = −0.032, *p* < .001, *z* = −3.782). However, this indirect effect was not significant for football event 1 (*b* = −0.039, *p* = .318, *z* = −0.998), the rugby (*b* = −0.052, *p* = .136, *z* = −1.493) or the horse racing (*b* = −0.027, *p* = .559, *z* = −0.584) events, not supporting *H3*.

Hypothesis 4a Shared social identity with attendees at the sporting events will be positively associated with expectations of support.

In support of *H4a*, higher shared social identification was associated with greater expectations of support at football event 1 (*b* = 0.326, *p* < .001), football event 2 (*b* = 0.257, *p* < .001), the rugby (*b* = 0.308, *p* < .001) and the horse racing (*b* = 0.361, *p* < .001).

Hypothesis 4b Seeing others adhere to the COVID‐19 safety measures will be associated with increased expectations of support.

In support of *H4b*, seeing others adhering to measures was associated with increased expectations of support from other attendees to keep safe at football event 1 (*b* = 0.440, *p* < .001), football event 2 (*b* = 0.512, *p* < .001), the rugby (*b* = 0.446, *p* < .001) and the horse racing (*b* = 0.522, *p* < .001).

Hypothesis 4c The relationship between seeing others adhere to the COVID‐19 safety measures and perceived risk of germ spread will be mediated by expected support from other attendees.

In support of *H4c*, there was a significant indirect effect of others’ adherence on reduced perceived risk via increased expected support at football event 1 (*b* = −0.117, *p* = .009, *z* = −2.612) and football event 2 (*b* = −0.158, *p* < .001, *z* = −8.264). However, this indirect effect was not significant for the rugby (*b* = −0.051, *p* = .192, *z* = −1.305) or the horse racing (*b* = −0.048, *p* = .438, *z* = −0.775), not supporting *H4c*.

Hypothesis 4d There will be a sequential mediation where seeing others adhere to the COVID‐19 safety measures will be associated with reduced perceived risk via increased shared social identity and increased expected support.

In support of *H4d*, there was a significant indirect effect of others’ adherence on reduced perceived risk via increased shared social identification and increased expected support at football event 1 (*b* = −0.042, *p* = .025, *z* = −2.246) and football event 2 (*b* = −0.020, *p* < .001, *z* = −5.378). However, this indirect effect was not significant for the rugby (*b* = −0.017, *p* = .188, *z* = −1.318) or the horse racing (*b* = −0.014, *p* = .479, *z* = −0.708), not supporting *H4d*.

#### Full model with the event as a covariate

**FIGURE 6:**
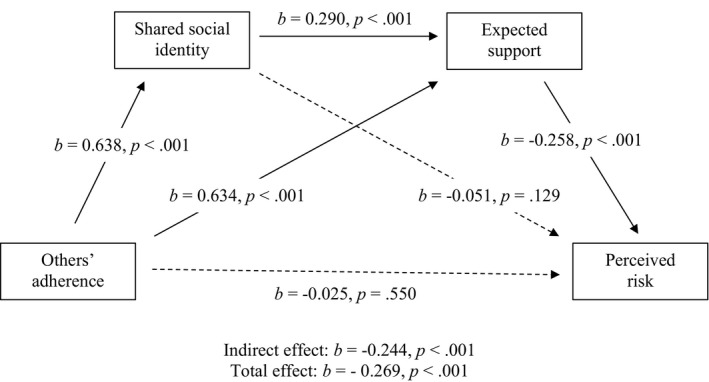
Sequential mediation model for all events

Finally, we tested the model including the data from all events and used event as a covariate. The full table and visualization of results can be found in Tables 9 and 10 and in Figure 6. There was no significant effect of seeing others adhere to the COVID‐19 safety measure on perceived risk, *b* = −0.025, *p* = .550, not supporting *H1*. In support of *H2*, there was a significant effect of seeing others adhere to COVID‐19 safety measures on increased shared social identity among attendees, *b* = 0.638, *p* < .001. Contrary to *H3*, there was no significant indirect effect on seeing others adhere to COVID‐19 safety measures on perceived risk via shared social identity with other attendees, *b* = −0.032, *p* = .131, *z* = −1.509. In support of *H4a*, there was a significant effect of increased shared social identity with attendees on increased expectations of support, *b* = 0.290, *p* < .001. In support of *H4b*, there was a significant effect of seeing others adhere to COVID‐19 safety guidance on increased expectations of support, *b* = 0.634, *p* < .001. In support of *H4c*, there was a significant indirect effect of seeing others’ adhering to COVID‐19 safety guidance on reduced perceptions of risk via increased expectations of support, *b* = −0.164, *p* < .001, *z* = −5.692. Finally, in support of *H4d*, there was a significant indirect effect of seeing others’ adhering to COVID‐19 safety guidance on reduced perceptions of risk via increased shared social identity and increased expectations of support, *b* = −0.048, *p* < .001, *z* = −5.381.

**TABLE 9:** Regressions for model with all events

	*b*	LCI	UCI	SE	*z*	*p*
OA–SSI	0.638	0.466	0.523	0.014	34.156	<.001
OA–ES	0.634	0.521	0.585	0.016	34.083	<.001
OA–PR	−0.025	−0.075	0.040	0.030	−0.598	.550
SSI–ES	0.290	0.286	0.369	0.021	15.664	<.001
SSI–PR	−0.051	−0.107	0.012	0.036	−1.516	.129
ES–PR	−0.258	−0.282	−0.140	0.036	−5.787	<.001
Age–SSI	0.050	0.014	0.072	0.015	2.917	.004
Age–PR	0.105	0.047	0.121	0.019	4.528	<.001
Event–SSI	−0.005	−0.060	0.044	0.027	−0.305	.760
Event–ES	0.020	−0.010	0.081	0.023	1.568	.117
Event–PR	0.106	0.082	0.226	0.037	4.185	<.001

Note

*b* = standardized beta coefficients; ES, expected support; LCI, lower confidence interval; OA, others’ adherence; PR, perceived risk; SE, standard error; SSI, shared social identity; UCI, upper confidence interval. All demographic variables were tested for significant associations with shared social identity, expected support and risk perception using standard error bootstrapped regressions with 10,000 samples. Only the significant demographics are included in the table and structural model.

**TABLE 10:** Total, direct and indirect effects for model with all events

	*b*	LCI	UCI	SE	*z*	*p*
Total effect	−0.269	−0.224	−0.157	0.017	−11.270	<.001
Direct effect	−0.025	−0.075	0.040	0.030	−0.598	.550
Indirect effects						
Total indirect effect	−0.244	−0.221	−0.126	0.024	−7.131	<.001
OA–SSI–PR	−0.032	−0.053	0.006	0.015	−1.509	.131
OA–ES–PR	−0.164	−0.156	−0.077	0.020	−5.692	<.001
OA–SSI–ES–PR	−0.048	−0.047	−0.022	0.006	−5.381	<.001

Note

*b* = standardized beta coefficients; ES, expected support; LCI, lower confidence interval; OA, others’ adherence; PR, perceived risk; SE, standard error; SSI, shared social identity; UCI, upper confidence interval.

Event was a significant covariate in predicting perceptions of risk (*b* = 0.106, *p* < .001), but not for shared social identity (*b* = −0.005, *p* = .760) or expected support (*b* = 0.020, *p* = .117).

## DISCUSSION

This study aimed to evaluate the role of social identity processes on perceived risk across a range of UK pilot sporting events held during COVID‐19, expanding the prior literature by evaluating the role of others’ adherence, shared social identity and expected support on perceived risk in a novel model within the extreme context of the pandemic.

It was hypothesized that seeing other spectators adhering to safety measures would be associated with reduced perceived risk via two mediators: increased shared social identity and increased expected support (*H4d*). Support for this hypothesis was found at both football events and for the model including all four events, but not at either the horse racing or rugby, demonstrating partial support for this hypothesis at only two of the events.

The support for the other hypotheses is also mixed. Across all events, seeing others adhere to the COVID‐19 safety guidance was associated with high shared social identity among spectators (*H2*), and shared social identification with attendees was associated with higher expectations that attendees would keep one another safe (*H4a*). Similarly, seeing others adhere to the COVID‐19 safety measures was associated with higher expectations of support at all events (*H4b*). However, the direct effect of others’ adherence on perceived risk (*H1*) was significant at all events except horse racing and the model including all events. Moreover, the relationship between seeing others adhere to the COVID‐19 safety guidance and reduced perceived risk was only mediated by shared social identity (*H3*) at football event 2. Finally, the relationship between seeing others adhere and perceived risk was only mediated by expected support (*H4c*) at the football events and for the model with all events, but not horse racing or rugby.

The results demonstrated a replicable association between observing other spectators adhering to safety measures and increased shared social identity across the four events. These results are in line with self‐categorization theory and the concept of normative fit (Turner et al., 1987), where observing behaviours consistent with the norms, values, and goals associated with a social category can increase a sense of shared social identity. Spectators at the pilot events observed others adhering to measures which demonstrated normative fit with behaviours in line with the goals and values of the group (Templeton et al., 2020, 2021). The present results suggest that the protective health behaviours operated as a demonstration of group membership and were associated with an increase in shared social identity. These results extend the prior literature by demonstrating how seeing others acting safely can increase a sense of shared social identity at crowd events and indicate an avenue for future research into how engagement in protective health behaviours can be seen as an indicator of group membership.

The consistent association between seeing other spectators adhering to safety measures and increased expected support from other attendees supports the prior literature on disasters, which demonstrates that observing social support is associated with an increase in expectations of support (e.g. Drury et al., 2016; Ntontis et al., 2018). Additionally, across all four events, shared social identity reported by spectators was associated with an increase in the support they expected from others. This association has been found previously in research on religious pilgrimages (Alnabulsi & Drury, 2014; Hopkins & Reicher, 2017; Pandey et al., 2014) and mass emergencies (Drury et al., 2016; Ntontis et al., 2018, 2020), but this is the first research to test to the association in a sports mass event context.

The findings of shared social identity on risk perception are varied. Previous research suggests that shared social identity can reduce perceived risk in multiple contexts (e.g. see Cruwys et al., 2021; Khazaie & Khan, 2019). However, the relationship between shared social identity and perceived risk was only significant at the football cup final. Relatedly, the relationship between seeing others adhering to the COVID‐19 safety measures and perceived risk was not mediated by shared social identity except at the football cup final. The significant result supports the prior literature; however, there are three events, and the model with all four events, where the association does not hold. The mixed support for the role of shared social identification in attenuating the perception that others can spread germs suggests that further work needs to evaluate the extent to which this relationship holds in different contexts, and the ways in which group membership can lead to risky or non‐risky behaviours that may impact perceived risk (e.g. in contexts where risk‐taking is normative as part of the event, see Pandey et al., 2014).

In addition, the relationship between seeing others adhering to the COVID‐19 safety measures was only significantly associated with reduced perceived risk via an increase in expected support for both football matches and for the model of all events. For the rugby and horse racing events, this mediation effect was not significant because increased expected support was not significantly associated with reduced risk perception. The mediation via expected support is in line with previous research indicating that expected support from in‐group members is associated with increased perceptions of safety at both religious pilgrimages (Alnabulsi & Drury, 2014) and music festivals (Drury et al., 2015), even when these events posed a real danger due to their highly dense environments. However, the non‐significant relationship between expected social support and perceived risk at rugby and horse racing raises questions about which particular characteristics of the event environments contributed to variations in this model. Future research could evaluate whether observing supportive behaviour can reduce perceptions of risk in other settings, and what specific behaviours may mitigate (or enhance) perceived risk.

Finally, the present study found a significant direct association between seeing other spectators adhering to COVID‐19 safety measures on reduced risk perception at both football events, and the rugby, but not for the horse racing or the model with all events (which can be explained by a full mediation via shared social identity and expected support). One possible explanation for this is that horse racing is the only entirely outdoor event and so participants may have perceived low risk regardless of others’ adherence. Indeed, government advice at the time of this event encouraged outdoor socializing compared with indoors due to reduced COVID transmission in open‐air environments (UK Government, 2020b). Additionally, interviews from the Events Research programme suggest that mask wearing was considered unnecessary by some participants in an outdoor environment (Templeton et al., 2021). The prior literature is limited in evaluating this direct effect. Thus, the results offer a greater insight into how observing safe, supportive behaviour may be associated with reduced risk perception, but that this is dependent on the event environment and whether the safe behaviours are perceived to be needed.

### Implications for theory and practice

Our findings offer several advances in theoretical understandings of social identity processes at mass events and implications for increasing safety at mass events.

The results of the present study offer unique insights into the effects of social identity processes on perceived risk because the test is replicated across four events in a unique context where the actual risk of COVID‐19 spread was very high in the United Kingdom. These unique data provide new theoretical implications to the mass event literature. First, it demonstrates that the theoretical perspectives—social identity theory and self‐categorization theory—are reinforced in a new social context, highlighting their potential applicability to further events. Second, it demonstrates that the processes previously described in the literature—observing supportive behaviour, shared social identity, expectations of support and risk perception—can be applied in a novel model, but that the relationships between the variables may be dependent on context and alternative factors not evaluated in the present or prior literature. For example, the mediating effects of shared social identity and expected support on risk perception were only found at the two football matches evaluated in this research, but not at the rugby or horse racing. This emphasizes the potential complexity of risk perception at sporting events and mass events more broadly. It demonstrates the need for further research into the factors associated with risk perception that have not been considered in the present research, alongside the replication of the present model in a range of mass event contexts.

It is also important to recognize the varying social identities and norms across the sporting contexts in the present study. For example, the collectivistic, social nature of football—with multiple social identities and associated social norms (for examples see Levine et al., 2005; Stott et al., 2001) contrasts with the individualistic nature of horse racing. Football attendees form distinct psychological crowds who support one of the two teams playing or are united to support the sport whereas attendees of horse racing events may be more individualistic because they are competing for individual horses in the race. The mean shared social identity for horse racing, however, did not differ from football, highlighting a potential limitation in item interpretation (see limitations section for further discussion).

Prior literature on mass events has demonstrated that both increased social identity processes and reduced risk perception are associated with a range of positive outcomes for attendees, both at the events and beyond (e.g. see Cruwys et al., 2019; Hopkins & Reicher, 2017; Khan et al., 2015; Tewari et al., 2012). This demonstrates the ‘social cure’ of mass events, which can be driven by the social identity processes evaluated in the present research (Jetten et al., 2017). From a business perspective, the increase in shared social identity found at all events and the reduction in risk perception found at some events in the present study suggests, alongside the prior literature, that spectators will purchase tickets for future sporting events (Silveira et al., 2018), even during COVID‐19.

Regardless of these business and psychological benefits, reduced *perceived* risk does not mean reduced *actual* risk. Indeed, literature has demonstrated that shared social identity, expectations of support and reduced risk perception can all be associated to an increase in risk‐taking behaviour at mass events (Hopkins & Reicher, 2017). For example, people prefer to be physically closer to in‐group members compared with outgroup members (Novelli et al., 2010) and may seek denser locations at crowd events because it is associated with a positive experience (Novelli et al., 2013), but close proximity in the current context can increase the risk of COVID‐19 transmission (World Health Organisation, 2020). Although the mediations evaluated in this study were not all significant, there was a consistent association between seeing others adhering and higher shared social identity at all four events. This highlights the potential for event organizers to harness social identities to facilitate safe behaviour rather than risky behaviour, such as showing how engaging in protective health measures is normative and expected by the group.

Support between in‐group members can be positive such as sharing practical resources in emergencies (see Drury et al., 2016), but they can also include high‐risk behaviours such as sharing razors for shaving heads at religious pilgrimages which can introduce routes for disease transmission (Hopkins & Reicher, 2017). Thus, it is important that event organizers understand what form supportive behaviours may take at events, and how shared social identities and expected support may lead to either safe or unsafe behaviours, as well as impact the perceived risk that others pose when that support is given (for more details see Templeton, 2021). For example, some season‐ticket holders at the football cup final removed their face masks and moved closer to other fans to show their support for the players together, and season‐tickets holders also reported lower perceived risk of COVID‐19 spread from others and higher comfort in close proximity with other attendees compared to non‐season‐ticket holders at the event (Templeton et al., 2021).

The present results and broader literature highlight how observing supportive behaviour, shared social identity and expected support can be used to promote health‐preserving behaviours, as these factors were all associated across the four events. However, future research must evaluate why the present model is only significant for perceived risk at the two football events, but not at horse racing or rugby. More accurate and effective interventions could be designed once a greater understanding of the processes associated with risk perception at these events are evaluated, beyond the consideration of shared social identity and expected support.

### Strengths, limitations and future research recommendations

The present study had an adequate sample size for all events except horse racing (*N* = 175), which may not have enough statistical power required to detect an effect. This could also explain the different results we found for horse racing compared to other events, specifically where no significant direct effect was found between seeing others adhering and perceived risk. Future research should replicate with larger sample sizes to determine the reliability of these findings before designing interventions accordingly.

This study was also limited in that there was no statistical method of accounting for the effects of time or environment, instead these were evaluated through seeing whether the mediation model would hold across the different events held in different environments and at different times. In addition, the present study could not evaluate this model within indoor events (such as nightclubs) where the risk of COVID‐19 transmission has been demonstrated to be higher through increased co2 levels in indoor environments (Department for Digital, Culture, Media, & Sport, 2021). We were also unable to compare results across different venue environments since the football 1, football 2 and rugby events all took place in open‐air theatres and the horse racing event (the only outdoor event) may have insufficient data for a reliable comparison.

Self‐categorization theory (Turner et al., 1987) highlights that salient social identities are context‐dependent; therefore, subordinate or superordinate identities can shift which behaviours are normative in any given context. The present study failed to consider whether subordinate identities—such as team identification—influenced the associations between others’ adherence and perceived risk via shared social identity and expected support, particularly at the football where these factors were significantly associated with reduced risk perception. Future research should evaluate the potential role of subordinate identities and should particularly consider the role of the salient social identity when designing interventions.

Additionally, it is unclear from the present study whether the attendee's definition of ‘the crowd’ varied between the different contexts. Rugby and football attendees often go in support of one of two teams, meaning items regarding shared social identity could be interpreted broadly as regarding all attendees, or as fans of the same team. Future research is required to evaluate how items related to the crowd are interpreted by attendees, particularly whether these vary across sporting contexts.

It is important to highlight that the present results are correlational and hence the relationships between these variables could reverse. For example, expected support may lead to increased shared social identification. Future research should, therefore, evaluate variations in this model to further evaluate the associations between these variables. The results are also limited in the quality of information they provide regarding the causes of the associations between others’ adhering, shared social identity and expected support across the four events. For example, although the present results support our hypothesis that observing others adhering to the COVID‐19 guidance would be associated with higher shared social identity, the findings do not tell us *why* it occurred. Future research should evaluate in more detail why the associations do or do not occur across different contexts, such as by using qualitative research to gain more nuanced data.

It must be acknowledged that participants may have developed COVID‐19 symptoms within the 15‐day response limit which could have impacted their responses. No data were collected regarding this to control for participants’ COVID‐19 status. However, evidence suggests limited spread of COVID‐19 at the Events Research Programme events, where the greatest proportion of positive cases were associated with mixing outside of the events themselves (Smith et al., 2021).

Additionally, our measures of perceived risk are retrospective, and so it is possible the present study is measuring support received rather than expected support, where interpretation of the items used could vary between participants. Future research should use clearer measures to ensure either expected support or received support is explicitly measured.

Finally, the present study design does not evaluate the behavioural impact of these social identity processes, particularly their influence on risk‐taking behaviours. It is only hypothesized from the prior literature that the social identity processes considered in the present research will increase risk‐taking behaviour. Thus, future research must evaluate behavioural data to test this hypothesis.

## CONCLUSION

The present study demonstrates that shared social identity and expected support mediate the relationship between others’ adherence and perceived risk at the football pilot sporting events held during COVID‐19, within the model including all events, but not at rugby or horse racing events. Specifically, it demonstrated that these social identity processes—shared social identity and expected support—are associated with observing other spectators adhering to measures and that shared social identification is associated with higher expectations of support from in‐group members. However, the sequential mediation models were not significant for rugby or horse racing. The results highlight the replicability of the prior literature in particular environments but highlight the need for further research to evaluate the influence of context and environment on models of risk perception.

## CONFLICT OF INTEREST

All authors declare no conflict of interest.

## AUTHOR CONTRIBUTION

**Kayleigh Smith:** Conceptualization; Data curation; Formal analysis; Methodology; Writing – original draft; Writing – review & editing. **Anne Templeton:** Conceptualization; Data curation; Formal analysis; Funding acquisition; Methodology; Supervision; Writing – review & editing.

## Data Availability

The data that support the findings of this study are openly available in the Open Science Framework at https://osf.io/25yw4.
